# The C.L.A.M.P. Nephrometry score: A system for preoperative assessment of laparoscopic partial nephrectomy with Segmental Renal Artery Clamping

**DOI:** 10.1038/s41598-018-28058-w

**Published:** 2018-06-26

**Authors:** Yichun Wang, Chen Chen, Chao Qin, Xiao Li, Yamin Wang, Jiayi Zhang, Yi Wang, Xiang Zhou, Qijie Zhang, Ninghong Song, Zengjun Wang

**Affiliations:** 1 Department of Urology, the First Affiliated Hospital of Nanjing Medical University, Nanjing, China; 2 Department of Urology, The affiliated Cancer Hospital of Jiangsu Province of Nanjing Medical University, Nanjing, China

## Abstract

Laparoscopic partial nephrectomy with segmental renal artery clamping is associated with a less warm ischemic injury and better postoperative affected renal function compared with main renal artery clamping. However, its indication remains unclear. We established a standardized nephrometry scoring system (The C.L.A.M.P. Nephrometry Score) to evaluate its flexibility in preoperative assessment. This scoring system based on 5 components. The ranking (C)oefficient of each score and the (L)ocation of the clamping position of the target artery and areas of the target artery entering the renal sinus: (A)nterior boundary, (M)ulti-boundary and (P)osterior boundary. We applied this system to analyze data from 106 consecutive patients who underwent SRAC during LPN and divided these patients into 3 groups based on their C.L.A.M.P. scores. The rate of conversion to main renal artery clamping and clamping success rate and the affected side GFR reduction showed significant differences among the groups (P < 0.001). However, parameters such as blood loss, Warm ischemia time and postoperative hospitalization were not significantly different. The C.L.A.M.P. nephrometry score shows strong ability in distinguishing different complexities of artery characteristics and plays a promising role in identifying patients who are suitable for the SRAC technique.

## Introduction

Renal cell carcinoma (RCC) is a malignant tumor of the urinary system. In recent decades, the incidence of this disease has increasing, with small renal carcinoma (smaller than 4 cm) constituting over a third of all cases^1,2^. The standard treatment for patients with small RCC is nephron sparing surgery (NSS). Since the first use reported by Winfield *et al*.^3^, laparoscopic partial nephrectomy (LPN) has gained worldwide popularity as a promising minimally invasive NSS for selected renal tumor^4–6^, and the development of NSS techniques is always focused on maximally reserving postoperative function^7^. Some novel techniques have emerged, includind zero ischemia^8^, zero ischemia with microdissection technique^9,10^, and segmental renal artery clamping (SRAC)^11^. Recently, minimizing warm ischemia injury has been a surgical focus and aims to maximize the preservation of nephron units, and the technique of segmental renal artery clamping has emerged as a promising method for renal hilar control^11^. Some studies have reported that SRAC is associated with decreasing warm ischemia^7,11–13^. SRAC appears promising in terms of reserving postoperative function.

However, the determination of when a patient is suitable for SRAC during LPN mainly depends on the individual experience of the surgeon, which is subject due to different training patterns and operation habits. Currently, many tumor complexity scoring systems have been proposed to quantify tumor characteristics. The most widely reported scoring systems are R.E.N.A.L, PADUA and the C-index, and several studies have revealed that these systems are associated with perioperative outcomes^2,14,15^. It appears that although these systems identify renal tumor complexity, none involves the arterial characteristics of the tumor. For patients undergoing SRAC before LPN, the anatomy of the segmental renal arteries is of great importance.

Inspired by these systems, we present a structured, quantitative scoring system (The C.L.A.M.P. nephrometry score) that aims to specifically describe the surgically relevant characteristics of renal tumor artery in SRAC and to evaluate its feasibility in preoperative assessment.

## Results

LPNs were completed in all cases without conversion to open or total nephrectomy. Of the cases in which SRAC was attempted, 21 were converted to main renal artery clamping due to uncontrollable bleeding. Other perioperative data are presented in Table 1.

**Table 1:** The perioperative demographic and clinical data of the patients.

Variable	Low	Medium	High	Over all	*P* value
Number of patients	56	44	6	106	—
Male, n (%)	35 (62.5)	24 (54.5)	3 (50.0)	62 (58.49)	0.660
Age, year	55.59 ± 13.08	56.41 ± 11.55	53.67 ± 7.09	55.82 ± 12.13	0.857
R.E.N.A.L score	6.52 ± 1.63	7.16 ± 1.61	7.00 ± 1.79	6.81 ± 1.65	0.148
Blood loss, mL	258.52 ± 185.17	302.50 ± 176.20	183.33 ± 65.01	272.52 ± 178.36	0.215
WIT, min	23.38 ± 5.55	25.55 ± 6.09	22.67 ± 4.08	24.24 ± 5.78	0.139
Postoperative Hospitalization, d	9.05 ± 3.20	8.18 ± 1.90	8.50 ± 0.55	8.66 ± 2.65	0.264
Main renal artery Clamping, n (%)	1 (1.79)	14 (31.82)	6 (100)	21 (19.81)	<0.001
Clamping success rate, %	99.40 ± 4.45	63.07 ± 16.71	41.94 ± 13.35	81.07 ± 23.15	<0.001

The rate of conversion to main renal artery clamping significantly differed among the groups (P < 0.001) and increased with the surgical complexity. The clamping success rate also differed among groups (P < 0.001). In the follow-up we only received 81 patients’ GFR data, the affected side GFR reduction percent among groups is distinct (low group: 24 ± 9 vs moderate group: 29 ± 13 vs high group: 32 ± 14, P < 0.001). However, the parameters including blood loss, warm ischemia time (WIT) and postoperative hospitalization, showed no significant difference (Table 1).

The C.L.A.M.P. nephrometry score system is based on 5 parameters including C, L, A, M and P, where C stands for the coefficient, L refers to the location of the clamping position, and A, M and P represent the areas where the TAFT enter the renal hilum, (Anterior boundary, Multi-boundary and Posterior boundary, respectively) (Table 2).

**Table 2:** The component of the C.L.A.M.P. Neprometry Score System.

Variable		No. 1	No. 2	No. 3
**(C)**oefficient		1	1/2	1/3
	Points	**1** **pt**	**2** **pt**	**3** **pt**
**(L)**ocation	X	≤1.1	1.2–1.5	≥1.6
	Y	≤0.5	0.6–0.7	≥0.8
		**Renal Hilar Approach Recommended**		
**(A)**nterior hemiboundary		Anterior hilar approach		
**(M)**ulti-hemiboundary		A Combination of the two approaches		
**(P)**osterior hemiboundary		Posterior hilar approach		
Complexity degree		Low	Moderate	High
C.L.A.M.P. score		[2–6)	[6–10)	≥10

The first variable of the scoring system is (C)oefficient. This is a particularly relevant factor when preoperatively considering the SRAC. If a tumor has more than one TAFT, the clamping process will be extended to some degree, and the complexity of the SRAC will increase with the number of TAFTs. TAFT was usually confirmed by physician carefully evaluating the CTA image. In our scoring system, if a tumor has more than one TAFT, each TAFT will be given a score based on the clamping location. The largest score is given a ranking coefficient of 1, the second is given a ranking coefficient of 1/2, and the third is given a ranking coefficient of 1/3.

**Figure 1:**
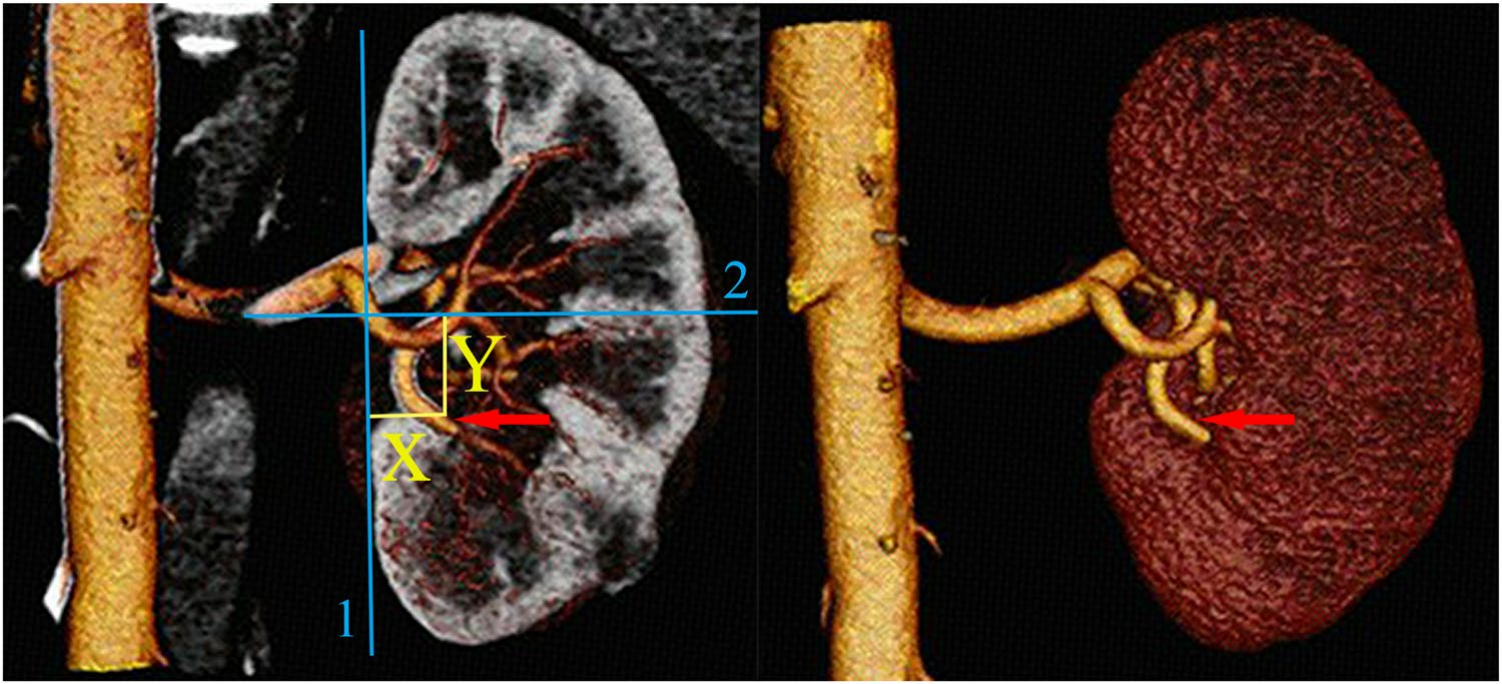
Preoperative assessment of the complexity of the SRAC based on a three-dimensional dynamic renal vascular model. Arrows show the target artery; Line 1 remains tangential to the ventral side of the kidney; Line 2 is in the midline of the kidney; X and Y refer to the distance from the clamping point to lines 1 and 2, respectively.

The second variable of the scoring system is (L)ocation. This variable focuses on clamping location, which is the most relevant characteristic when radiographically evaluating the complexity of SRAC. This variable contains two detailed parameters, X and Y (Fig. 1). First a straight line is drawn against the ventral side of the kidney and tangent to the upper and lower side of the kidney at the same time in the CTA image. Then, another line which crossing the renal hilum and perpendicular to the renal axis is introduced. The parameter X is measured as the maximal distance in any single plane of CTA image from the clamping position to the first line. For clarity, X refers to the depth of the clamping point inside the kidney. Based on the equation describing the clamping assurance of a certain artery based on our previous research and complex calculations^16^, 1 point is awarded for a score of X from 0 cm to 1.1 cm, 2 points are awarded for a score of 1.2 cm to 1.5 cm, while 3 points are awarded for a distance more than 1.6 cm. Similarly, Y refers to the distance from the clamping position to the renal midline. If the value of Y is 0.8 cm or more, 3 points are awarded, values between 0.6 cm and 0.7 cm are assigned 2 points, and distances within 0.5 cm are assigned 1 point. It is important to emphasize that X and Y may not be identified based on a single image but should be evaluated in different images, and the maximal value should be recorded.

**Figure 2:**
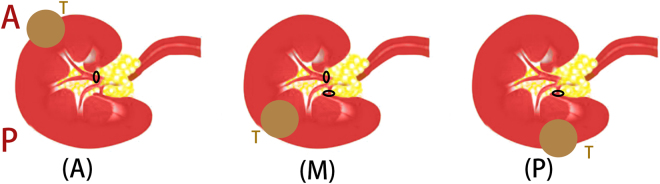
Different patterns recommended for the choice of renal hilar approach. A: anterior; P: posterior; T: tumor; (A): anterior hemiboundary; (M): Multi-hemiboundary; (P): posterior hemiboundary; The black ellipse represents the clamping position.

The last three variables focus on the approach from where the targeted artery enters the renal sinus (Fig. 2). As mentioned and advocated previously^13^, different surgical approaches should be adopted to dissect different target arteries: the anterior hilar approach, the posterior hilar approach and a combined approach. The choice of the approach used is mainly based on the anatomical characteristics of the target. All arteries entering the renal are distributed around the sagittal boundary of the renal sinus. TAFTs entering the (A)nterior hemiboundary should be operated on using an anterior hilar approach, whereas the TAFTs entering the (P)osterior hemiboundary should be operated on using the posterior hilar approach. If the tumor is supported by (M)ulti-hemiboundary TAFTs, a combination of the two approaches should be adopted.

Table 2 summarizes the 5 components of the C.L.A.M.P. nephrometry scoring system. According to clinical experience and statistical analysis, a scoring equation was developed:

$${\rm{C}}.{\rm{L}}.{\rm{A}}.{\rm{M}}.{\rm{P}}.\,{\rm{Score}}={({\rm{X}}+{\rm{Y}})}_{1}\,\ast 1+{({\rm{X}}+{\rm{Y}})}_{2}\,\ast 1/2+\ldots {({\rm{X}}+{\rm{Y}})}_{{\rm{x}}}\,\ast 1/{\rm{x}},$$

where (X + Y)_x_ stands for the ranking number of the TAFT. If there is only one TAFT, the remaining terms (L_2_, L_3_…) should be neglected.

We retrospectively analyzed the clinical data of consecutive patients who underwent SRAC at our institution; the patients were divided into three groups according to the C.L.A.M.P. score. Patients with low complexity more often underwent SRAC and achieved a higher clamping success rate than patients in the moderate complexity group. Patients in the high complexity group were more likely to require main artery clamping during the surgery. The perioperative data of patients in the three groups were collected and compared (Table 1).

**Figure 3:**
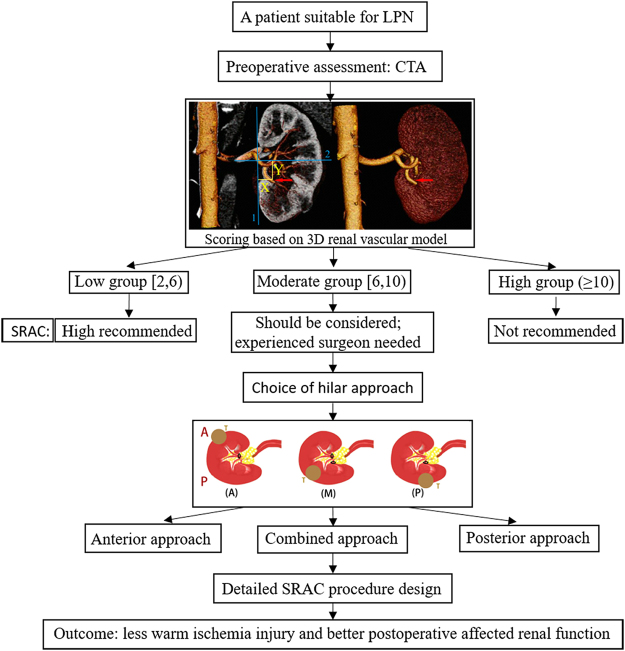
The management of potential patients for the SRAC procedure.

A flowchart was developed for surgeons to better understand how to apply this scoring system (Fig. 3).

## Discussion

Since the SRAC technique was first proposed by Shao *et al*. in^11^, SRAC has proved successful in converting global parenchymal ischemia to regional ischemia, which can minimize warm ischemia and provide better early postoperative renal function compared with traditional clamping. Early studies have demonstrated some key points of this surgery. After the introduction of SRAC^11^, Shao *et al*. also developed the precise clamping skill used under the guidance of dual-source computed tomography angiography, which made the technique more mature^12^. In addition, they also established a standard renal hilar approach for SRAC to standardize the surgery^13^. In 2016, a model for assuring the clamping success of SRAC was developed^16^. This model is beneficial for the surgeon while designing an SRAC program and can distinguish the clamping artery that provides high assurance from those providing low assurance and can help surgeons to choose a suitable artery for clamping. Existing studies on the development of the SRAC technique have solved the operational details. Nevertheless, some important questions remain, such as the indications for using this promising technique and how to evaluate the complexity of SRAC preoperatively.

Early in 2009, Alexander Kutikov and Robert G. Uzzo proposed the first nephrometry scoring system^2^. Since then, several scoring systems have been described with the aim of obtaining quantitative data about the characterization of renal tumor anatomical elements before surgical decisions are made. In clinical practice, these systems appear to differ in their ability to predict the complexity of a surgery and perioperative complication rates^2,14,15^. Different from these systems, our C.L.A.M.P. scoring system focuses on the characteristics of the target artery instead of the tumor itself. The system consists of two quantitative parameters and three descriptive parameters. It can identify the patients suitable for SRAC technique and provide the surgeon with necessary information on how to choose an appreciate renal hilar approach.

The first variable of the scoring system is (C)oefficient. This is a particularly relevant factor when preoperatively considering SRAC. During the routine preoperative evaluation, the tumor is considered based on a three-dimensional dynamic renal vascular model. If the tumor is supplied by more than one target artery, the complexity of the surgery is increased; the complexity further increases as the number of the feeding arteries increases. However, it is worth noticng that each TAFT makes a different contribution to the surgery. The complexity of the surgery mostly depends on the artery that is the hardest to address. In our scoring system, each TAFT is given a score based on the characteristic of the TAFT, and each score will also be given a coefficient. The largest score will be given a ranking coefficient of 1, the second a coefficient of 1/2, and the third a coefficient of 1/3. For ease of understanding, arteries with different coefficients contribute different degrees of complexity to the surgery. For example, suppose that a tumor has three feeding arteries, scored 5, 4 and 3. The first artery has the modified score 5 * 1 = 5, the second has the modified score 4 * 1/2 = 2, and the last has the modified score 3 * 1/3 = 1. Thus, the final score is 5 + 2 + 1 = 8. We can easily see that the artery with the largest score plays the greatest part in determining the final score. However, the other target arteries transform the surgery from one of low complexity (score 5) into one of moderate complexity (score 8).

The second variable of the scoring system is (L)ocation. This variable describes the location of the clamping position; before using this system, the target artery and it’s clamping position should be identified on the computed tomography arteriography image. The protocol used to identify the clamping position has been described in our previous research^16^. This variable contains two parameters, X and Y. X refers to the depth of the clamping point inside the renal, and larger values of X tend to increase the difficulty of clamping; Y refers to the distance from the clamping position to the kidney midline. In this system, higher scores indicate that the clamping point is far from the kidney midline. In clinical practice and during the development of this system, we observed that independent of the value of X and Y, the clamping procedure will be more complex when the clamping point is far from the dissociative renal hilar. To explain this, we hypothesize that the surgeon’s view and the operating space are responsible; this reminds us of the importance of port distribution. It is easy to understand that the distribution of ports can determine the operating experience by providing good vision and leaving enough space for the operation; sometimes, a poor port distribution can make the operation extremely difficult. This hypothesis was validated by Shao *et al*., and we also advocate three alternative approaches for the management of target arteries with different distributions^13^.

The last three variables focus on the approach from where the targeted artery enters the renal sinus. They can provide the surgeon with the message on how to choose a renal hilar approach. As mentioned above, the choice of surgical approach is mainly based on the anatomical characteristics of the targets. TAFTs entering the (A)nterior hemiboundary should be operated on using the anterior hilar approach, whereas the TAFTs entering the (P)osterior hemiboundary should be operated on using the posterior hilar approach. If the tumor is supported by (M)ulti-hemiboundary TAFTs, a combination of the two approaches should be adopted.

Different from several other tumor scoring systems, the C.L.A.M.P. scoring system does not include the size of the tumor. Tumor size has been established as a strong prognostic indictor for surgical outcome in radical or partial nephrectomy. However, this scoring system was designed only to evaluate the complexity of the SRAC procedure and does not refer to the following tumor excision process.

Whereas the aim of the C.L.A.M.P. scoring system is to introduce quantitative information regarding the targeted renal artery, the individual components of the system score are added to form a sum. For example, cases with only one TAFT, where the clamping point is 1.5 cm from the renal hilum and 0.6 cm from the kidney midline and posterior hemiboundary are scored (2 + 2) * 1 + p = 4p. Based on simple calculations, the characteristics of the target artery and the complexity of the surgery can be evaluated using this standard scoring system perioperatively.

The warm ischemia time, interoperative bleeding and postoperative hospitalization did not differ among three groups. It is worth noticing that the warm ischemia time do not equate with affected renal warm ischemia injury. Apart from warm ischemia time, warm ischemia area may also contribute to the whole affected renal warm ischemia injury. SRAC could convert global parenchymal ischemia to regional ischemia. As our earlier study shows, the percentage of preserved parenchyma volume may play a more prominent role in postoperative renal function. This may account for the similar warm ischemia time and the distinction affected GFR reduction. The warm ischemia time, interoperative bleeding and postoperative hospitalization are associated with the surgery LPN. According to many researchers^17,18^, the tumor characteristics may account for this. In our research, the R.E.N.A.L score did not differ among the three groups; in other words, the tumor anatomy among groups is similar.

The rate of conversion to main renal artery clamping and the clamping success rate differed significantly among the groups (P < 0.001). indicating that the C.L.A.M.P. neprometry score system successfully distinguished the patients in the three groups according to surgical complexity. and identified the patients suitable for SRAC surgery. The low complexity group achieved a high clamping success rate (99.40%) and low rate of conversion to main renal artery clamping (1.79%). These patients, can benefit from SRAC surgery. For surgeons who intend to start using this technique, these patients should be considered the best candidates. The patients in the moderate complexity group achieved a higher failure rate than those in the low complexity group; however, according the affected renal function, most of them still benefited from this technique because of its lower rate of intraoperative warm ischemia injury and its better early postoperative affected renal function compared with main renal artery clamping. However, for surgeons unfamiliar with this technique, these cases are not suitable. We do not recommend that patients in the high complexity group undergo this demanding procedure. An ideal classification system should be simple in its application and provide enough information about the surgery. Furthermore, for use as a preoperative scoring system, convenience should also be considered an important factor in the application of this system. We developed a flowchart for the management of patients who may be suitable for the SRAC procedure (Fig. 3).

In the C.L.A.M.P. scoring system, we proposed the use of 5 associated parameters for the SRAC procedure. However, other factors might affect the operation, and the detailed scoring boundary of X and Y is defined by the association of the distance and the clamping assurance. We cannot exclude the possibility that a better boundary or other parameters with a stronger distinguish ability exists. Our study was carried out at a single center, and verification at other institutions is essential.

The C.L.A.M.P. scoring system is the first scoring system to describe the surgically relevant characteristics of renal tumor artery in SRAC. This system successfully distinguishes different characteristics that are associated with arterial complexity and divides cases into three complexity groups. This system plays a promising role in the preoperative assessment of patients and can identify patients who are suitable for SRAC.

## Methods

### Study design and clinical data

The study was approved by the Institutional Review Board of the First Affiliated Hospital of Nanjing Medical University. All methods were performed in accordance with relevant guidelines and regulations. From December 2009 to June 2016, the clinical data of 106 consecutive patients who underwent SRAC during LPN were analyzed. Routine preoperative written informed consent and written informed consent for the publication of clinical information and images were obtained from all patients involved in this study. All patients underwent preoperative computed tomography angiography (CTA) routinely to establish a 3D model and to delineate the characteristics of the renal vasculature. The detailed imaging procedure applied in our institution has been described in our early study^12^. Perioperative data were collected and are presented in Table 1. The inclusion criteria for LPN were a single, organ-confined mass of ≤4 cm. Patients with a slightly large tumor (>4 cm) were also included if LPN was considered technically feasible. The contralateral kidneys of all patients were normal.

Split renal function was evaluated before and 3 months after the operation. The GFR was obtained using a camera-based method measuring the renal uptake of technetium-99 m diethylenetriaminepentaacetic acid (Gates’ method).

### Prediction of the accurate clamping position

The tumor was often supplied by secondary or tertiary branches of the main renal artery. To minimize the ischemic area and avoid unnecessary ischemia of normal parenchyma, the branch should be clamped where it has no further bifurcation before entering the parenchyma. Usually, this clamping position is close to the hilar parenchyma. The accurate clamping position was presented in the vasculature model (Fig. 1).

### Surgical procedure

The procedure of SRAC before LPN and three guiding rules for the clamping program have been described in detail in our early research^11–13,16^. First, patients were administered general anesthesia and placed in the lateral decubitus position. Then, four ports were constructed in the lumbar region. After the perirenal fat surrounding the tumor was removed and the renal artery was isolated, the target segmental renal arteries were identified and clamped with a bulldog clamp. The correct clamping position is usually close to the hilar parenchyma as mentioned preciously^13^. After successfully clamping the target segmental renal arteries, renorrhaphy was performed with the tumor excised closely around its capsule together with a margin of 1–2 mm of normal parenchyma. Successful clamping meant that the target artery was blocked without blood flow. If the target artery failed to be isolated, this clamping procedure was unsuccessful. In such situations, main artery clamping was considered when there was excessive bleeding without enough time left to close the defect.

### Development of the C.L.A.M.P. nephrometry score

Renal vasculature characteristics that might affect the SRAC were collected through a preoperative radiological assessment. The following 5 critical artery parameters were carefully discussed: the ranking (C)oefficient of each score, the (L)ocation of the clamping position of the target artery feeding the tumor (TAFT) and the areas of the target artery entering the renal sinus: (A)nterior boundary, (M)ulti-boundary and (P)osterior boundary. An equation involving the clamping assurance of a certain artery was used in the development of the scoring system. This equation has been described in our previous report^16^.

### Statistical analysis

State 12.0 was used for all analyses. Pearson’s chi-squared test for continuous and categorical variables was performed to evaluate differences in the perioperative data. P values < 0.05 were considered significant.

### Availability of materials and data

Written informed consent for publication of their clinical details and clinical images were obtained from all patients. The datasets supporting the conclusions of this article are available from the First Affiliated Hospital of Nanjing Medical University (Nanjing, Jiangsu, China). Please contact the corresponding author for data requests.
